# XRCC4 and MRE11 Roles and Transcriptional Response to Repair of TALEN-Induced Double-Strand DNA Breaks

**DOI:** 10.3390/ijms23020593

**Published:** 2022-01-06

**Authors:** Ronald Benjamin, Atoshi Banerjee, Xiaogang Wu, Corey Geurink, Lindsay Buczek, Danielle Eames, Sara G. Trimidal, Janice M. Pluth, Martin R. Schiller

**Affiliations:** 1 Nevada Institute of Personalized Medicine, University of Nevada Las Vegas, Las Vegas, NV 89154, USA; atoshibanerjee@gmail.com (A.B.); shorewoo@gmail.com (X.W.); coreygeurink@gmail.com (C.G.); buczek@unlv.nevada.edu (L.B.); eamesdani@gmail.com (D.E.); saratrimidal@gmail.com (S.G.T.); 2 School of Life Science, University of Nevada Las Vegas, Las Vegas, NV 89154, USA; 3 Health Physics and Diagnostic Sciences, University of Nevada Las Vegas, Las Vegas, NV 89154, USA; janice.pluth@unlv.edu

**Keywords:** DNA repair, TALEN, NHEJ, end joining, XRCC4, mirin

## Abstract

Double-strand breaks (DSB) are one of the most lethal forms of DNA damage that, if left unrepaired, can lead to genomic instability, cellular transformation, and cell death. In this work, we examined how repair of transcription activator-like effector nuclease (TALEN)-induced DNA damage was altered when knocking out, or inhibiting a function of, two DNA repair proteins, XRCC4 and MRE11, respectively. We developed a fluorescent reporter assay that uses TALENs to introduce DSB and detected repair by the presence of GFP fluorescence. We observed repair of TALEN-induced breaks in the *XRCC4* knockout cells treated with mirin (a pharmacological inhibitor of MRE11 exonuclease activity), albeit with ~40% reduced efficiency compared to normal cells. Editing in the absence of XRCC4 or MRE11 exonuclease was robust, with little difference between the indel profiles amongst any of the groups. Reviewing the transcriptional profiles of the mirin-treated *XRCC4* knockout cells showed 307 uniquely differentially expressed genes, a number far greater than for either of the other cell lines (the HeLa *XRCC4* knockout sample had 83 genes, and the mirin-treated HeLa cells had 30 genes uniquely differentially expressed). Pathways unique to the *XRCC4* knockout+mirin group included differential expression of p53 downstream pathways, and metabolic pathways indicating cell adaptation for energy regulation and stress response. In conclusion, our study showed that TALEN-induced DSBs are repaired, even when a key DSB repair protein or protein function is not operational, without a change in indel profiles. However, transcriptional profiles indicate the induction of unique cellular responses dependent upon the DNA repair protein(s) hampered.

## 1. Introduction

DNA carries genetic instructions for the development and function of all known living organisms; therefore, it is important to preserve the integrity of the DNA. However, DNA is not inert, but susceptible to multiple types of damage. The common sources of DNA damage include environmental agents such as UV light, ionizing radiation, and chemical mutagens. Additionally, endogenous biological processes such as cellular metabolism including oxidative damage, DNA alkylation or hydrolysis, and double-strand breaks (DSBs) from collapsed replication forks contribute to DNA damage. Every day, DNA in normal cells has approximately 10–50 DSBs and thus requires efficient repair of DNA damage to maintain its integrity [1]. Failure to repair such damage can lead to genomic instability, cellular transformation, and cell death. 

To mitigate DNA damage and maintain integrity, cells have multiple molecular mechanisms to repair different types of damage. The most deleterious DNA damage is arguably a DSB, which, if left unrepaired, threatens the loss of chromosomal content. The key machinery to repair DSBs are: (i) classical non-homologous end joining (referred to herein as NHEJ) repair, and (ii) homologous recombination repair (HRR) [2]. Studies using chicken DT40 cells deficient in NHEJ or HRR proteins established NHEJ as the predominant form of repair in G1 phase, able to commence repair in any phase of the cell cycle, whereas HRR was active only during late S or G2 phases of the cell cycle [3]. NHEJ is catalyzed by two core protein-DNA complexes, Ku70/Ku80/DNA-PKcs and DNALig4/XRCC4/XLF [4]. The heterodimer Ku70/80 is first to recognize the DSB ends, allowing the DNA-dependent protein kinase catalytic subunit (DNA-PKcs) to then load onto the ends acting as a scaffold for the assembly of the remaining NHEJ factors [5]. In G1, a mammalian cell can repair the majority of its DNA DSBs within ~30 min using NHEJ [2]. However, a biphasic repair of DNA DSBs has been observed with a half-time for fast repair of between 7 and 14 min, and the half-time of slow repair (~15% of breaks) between 60 and 90 min. The fast process is not thought to require resection, whereas the slow repair process does [2]. Typically, NHEJ attempts to repair DNA DSB first, but if a cell is in late S or G2 phases, it will be processed by HRR at a later time following DNA damage [6,7]. One disadvantage of NHEJ is that it is slightly more error prone and often introduces indels or scars [8], whereas HRR is less error prone in comparison [9].

XRCC4 is a well-studied DNA repair protein known to stimulate the activity of DNA ligase IV, catalyzing DNA end joining [10]. Schulte-Uentrop et al. demonstrated that knockout of *XRCC4*, a component of the LIG4/XLF/XRCC4 complex, rendered cells sensitive to DNA damage caused by either ionizing radiation or enzymatic cleavage of genomic DNA such as the TALENs we use in this paper [11,12]. Targeted inhibition of XRCC4, a component of the NHEJ ligation complex, should inhibit standard NHEJ-mediated DNA repair [8,9]. However, other backup repair pathways besides HRR do not require XRCC4. For example, an alternative form of DSB repair called alternative-end joining (A-EJ) was identified from persistent end joining activity in cells deficient for NHEJ [13]. A-EJ is evolutionarily conserved and can act in both NHEJ-proficient or -deficient cells [14]. A-EJ can mediate repair by three different pathways [15]. Single-strand annealing (SSA) requires an end resector of either MRN/CtIP/EXO1/DNA2, Rad52 as an end bridger, and XPF-ERRC1 endonuclease for flap cleavage. Microhomology-dependent DNA repair (MMEJ) requires one of the resectors listed in SSA as well as PARP, MRN or polθ as an end bridging factor. Finally, end-joining (EJ) requires PARP, an end-bridger, and ligase I as a ligating factor [16]. The A-EJ pathways are thought to be highly mutagenic, with MMEJ using short sequence homologies near the two ends and often resulting in small deletions [17]. In addition, the loss of XRCC4 expression increased the dependence of repair on the MMEJ repair pathway [18]. 

MRE11 performs its functions as part of the MRN complex, consisting of three proteins: meiotic recombination 11 protein (MRE11), RAD50, and Nijmegen breakage syndrome 1 (NBS1; also known as nibrin). MRE11 has two different nuclease activities: an endonuclease activity that is essential to initiate DNA end resection, and a 5′ to 3′ exonuclease activity. MRE11 was first noted to have a role in HRR, with subsequent work revealing additional roles for MRE11 in NHEJ and A-EJ [19,20]. Its function in NHEJ and HRR is thought to be mainly as a scaffold to help aid in DNA synapsis, whereas, its nuclease is key in its role in A-EJ, specifically MMEJ repair [21]. Mirin (*Z*-5-(4-hydroxybenzylidene)-2-imino-1,3-thiazolidin-4-one) inhibits Mre11-associated exonuclease activity, preventing MRN-dependent activation of ATM without affecting ATM protein kinase activity, eliminating the G2/M checkpoint and HRR in mammalian cells [22]. Depletion of MRE11 is associated with the reduction in MMEJ repair in normal cells and inhibition of resection in HeLa XRCC4 knockout cells, which further supports its role in A-EJ [19].

DSB repair has been extensively studied by damaging DNA in a GFP reporter harboring a I-Sce1 meganuclease cleavage site [23]. In the past decade, new gene-editing approaches such as zinc finger nucleases (ZFNs), transcription factor-like effector nucleases (TALENs), and clustered regularly interspaced short palindromic repeats (CRISPR)/Cas9) have emerged and gained widespread applications in basic science, biotechnology, and medicine. However, despite growing applications, far less is known about the repair pathways and proteins functioning to repair damage induced by TALENs. TALENs bind to DNA through a DNA binding domain consisting of an array of modular repeats with each repeat binding a specific base in the DNA target. These TALEs are fused to the FokI endonuclease catalyzing DSBs upon dimerization. Mosbach et al. showed that recombination proteins Rad52, SAE2, and MRE11-Rad50 complex are required for repair of DSB induced by TALENs in yeast [24]. Our lab had previously used TALEN-based editing to inactivate HIV proviral DNA [25], and have now adapted this system to study the repair mechanism(s) that catalyze repair of TALEN-induced DSBs. 

In this work, we constructed a TALEN-based reporter assay system that can detect repair following TALEN-induced DSBs. Using this reporter construct, we show that repair occurs even when both the XRCC4 protein and MRE11 exonuclease activity are inhibited separately, or simultaneously. Furthermore, the transcriptional profiles of the HeLa *XRCC4* knockout cells treated with mirin, a pharmacological inhibitor of MRE11 exonuclease activity, had 307 uniquely differentially expressed genes, many more transcripts than either the HeLa-NT (control), *XRCC4* knockout HeLa or mirin-treated HeLa cells. The inhibition of XRCC4 and MRE11 exonuclease activity was associated with differential expression of p53 downstream pathways, and metabolic pathways suggesting adaptation of the cells to target regulation of energy and stress upon DNA damage and inhibited repair. 

## 2. Results

### 2.1. Construction and Validation of a DNA Repair Reporter Assay

**Figure 1:**
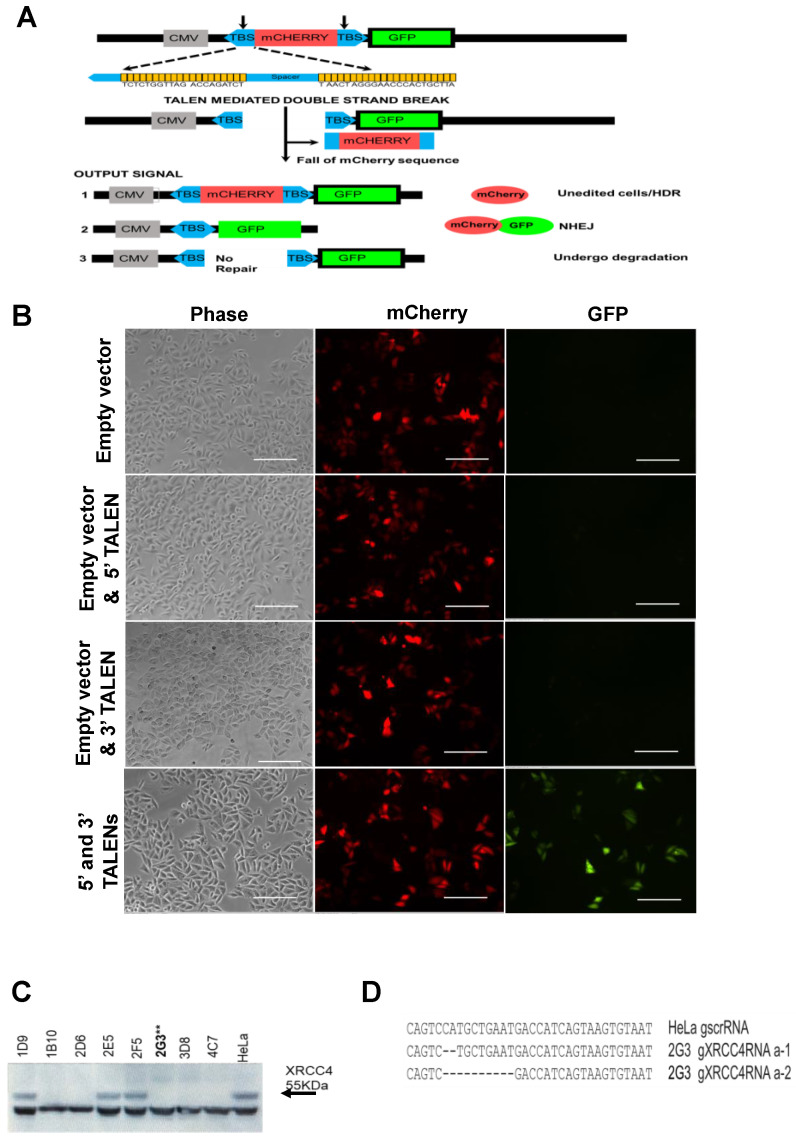
Construction and validation of the repair reporter system. (**A**) Schematic representation of reporter system and plausible outcomes. Outcome signal 1 produces mCherry fluorescence from plasmids that are not edited or are repaired by homology-directed repair (HDR), splicing an unedited fragment from another copy of the plasmid in the cell. Note that for output signal 2, both mCherry and GFP fluorescence would occur because during transient transfection only a fraction of transfected copies of sequences will likely be spliced to eliminate mCherry and express GFP. For output signal 3, if the construct remains unrepaired no fluorescence will be produced for GFP or mCherry. (**B**) Validation of reporter system by fluorescent microscopy identifies transfected mCherry positive cells and reveals repair-induced GFP protein expression when both TALENs are co-expressed. Representative images from triplicate experiments are shown. (**C**) Western blot confirmation of clonal selection of *XRCC4* knockout from HeLa cells in clone 2G3** revealed no XRCC4 expression. ** indicates clone used for experiments. (**D**) The *XRCC4* gene sequence corresponding to the target region in the 1st exon (nucleotide position 179–212th) of *XRCC4* for wild-type cells and *XRCC4* knockout 2G3 clone. Deletions are indicated by “-”. Clone 2G3 contains a frameshift indel on all alleles of XRCC4. Abbreviations: TBS = TALEN binding site; CMV = cytomegalovirus promoter. Scale bar = 100 μm.

To test and detect DNA repair following TALEN-induced DSBs, we first constructed and tested a plasmid encoding an extrachromosomal reporter assay system. The reporter plasmid has a CMV promoter for constitutive expression of the mCherry coding sequence. The mCherry coding sequence is flanked on either side with TALEN binding sites [TBSs, (Figure 1A)]. Under normal conditions, the mCherry reading frame terminates with a stop codon. Consequently, the downstream GFP coding sequence lacks a promoter; and hence is not expressed. When the cells are co-transfected with the reporter plasmid and the pair of TALEN-expressing plasmids that target the encoded TBSs, DSBs are introduced at both TBSs, thus excising the mCherry coding region. Upon subsequent repair, the CMV promoter is ligated in proximity to the GFP coding region, driving its expression. Therefore, GFP is expressed upon repair of the TALEN-induced DSBs. A flow chart depicting the relationships between various DNA editing possibilities and fluorescence output is shown (Figure 1A). 

First, we tested the reporter assay system in HeLa cells. The reporter plasmid was co-transfected with TALEN expression constructs T256 and T278, which recognize the 5′ and 3′ sequences, respectively. GFP positive (GFP^+^) cells were detected indicating editing (Figure 1B). Co-transfection of the reporter plasmid with empty vectors, or with the 5′ or 3′ TALEN from the pair did not express any GFP^+^ cells as would be expected for cells that are unedited or mono-edited (Figure 1B). This experiment confirms that our assay system is functional, sensitive, detects DNA repair, and requires both the 5’ and 3’ TALENs. 

### 2.2. Inhibition of MRE11 Exonuclease Activity in an XRCC4-Deficient Background

To determine the effect of loss of *XRCC4* and MRE11 exonuclease function on TALEN-induced repair, we needed to abolish or inhibit this protein or protein function, respectively. *XRCC4* was knocked out in Hela cells using targeted CRISPR-Cas9 editing within the coding region. The *XRCC4* knockout in clone 2G3 was confirmed by the loss of XRCC4 protein expression as detected by Western blot analysis (Figure 1C). A complete absence of XRCC4 protein expression was observed, and this clone was selected for further experiments. The knockout in clone 2G3 was confirmed by Sanger sequencing and revealed a 2 bp deletion and 10 bp deletion in each allele, respectively, of the *XRCC4* gene (Figure 1D). For knockout of the MRE11 exonuclease function, we used mirin, which has been published to inhibit MRE11 exonuclease and other downstream activities at a concentration of 100 μM [22,26,27,28,29,30]. 

### 2.3. XRCC4-Deficient Cells Treated with Mirin Still Repair TALEN-Induced Breaks

**Figure 2:**
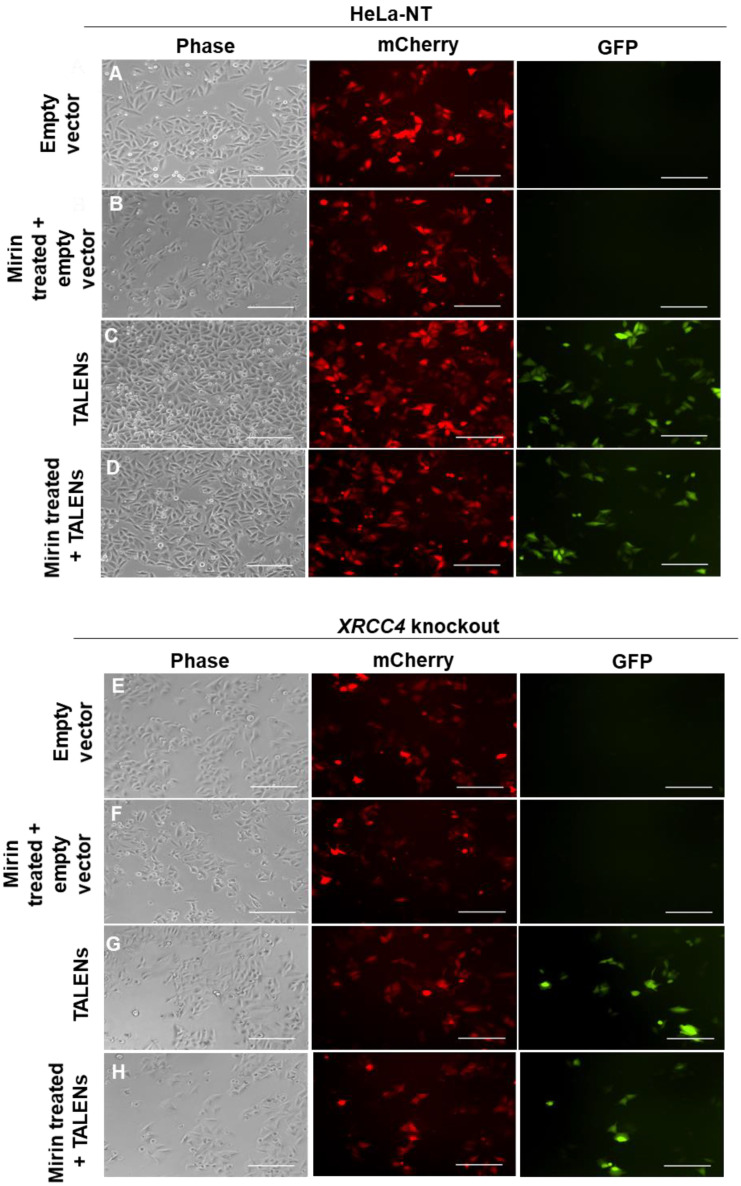
DNA DSB repair when either *XRCC4* is knocked out, MRE11 is inhibited, or both. Representative fluorescence microscopy images of cells following various treatments (treated with 100 μM mirin where indicated) for HeLa-NT (**A**–**D**) and XRCC4 knockout (**E**–**H**) cells. Cells co-transfected with a TALEN reporter plasmid (expressing mCherry) along with either empty vector or TALEN plasmids produced GFP^+^ cells indicating DNA repair editing had occurred. Cells were analyzed 48 h post-transfection and representative images are shown. TALEN reporter with empty vector (**A**,**E**), empty vector with mirin (**B**,**F**), TALEN plasmid (**C**,**G**), TALEN plasmid with mirin (**D**,**H**) are shown. Scale bar = 100 μm. Representative images from triplicate experiments are shown.

TALENs have a high affinity (nM) for DNA, which could interfere with DNA repair [31]. We assessed whether TALENs impact DNA NHEJ repair activity. *XRCC4* knockout and control cells HeLa-NT (non-targeting) transfected with reporter plasmid and empty TALEN vectors did not show any GFP^+^ cells as expected for these negative controls (Figure 2A,E). Mirin treatment of these cells also did not affect GFP expression (Figure 2B,F). mCherry expression indicated that the cells were expressing the reporter construct (Figure 2). Many HeLa-NT and *XRCC4* knockout cells co-transfected with the reporter and TALEN constructs (T270 and T278) showed GFP expression, indicating DSBs followed by NHEJ repair (Figure 2C,G).

In co-transfected HeLa-NT cells treated with mirin, GFP^+^ cells were present indicating repair when MRE11 exonuclease activity is inhibited (Figure 2D). Surprisingly, co-transfected mirin-treated *XRCC4* knockout also showed repair as indicated by the presence of GFP expression (Figure 2H). This suggests that repair is sustained even when *XRCC4* is knocked out and MRE11 exonuclease activity are inhibited.

### 2.4. Repair Efficiency Is Lowered in XRCC4-Deficient Cells Treated with Mirin

**Figure 3:**
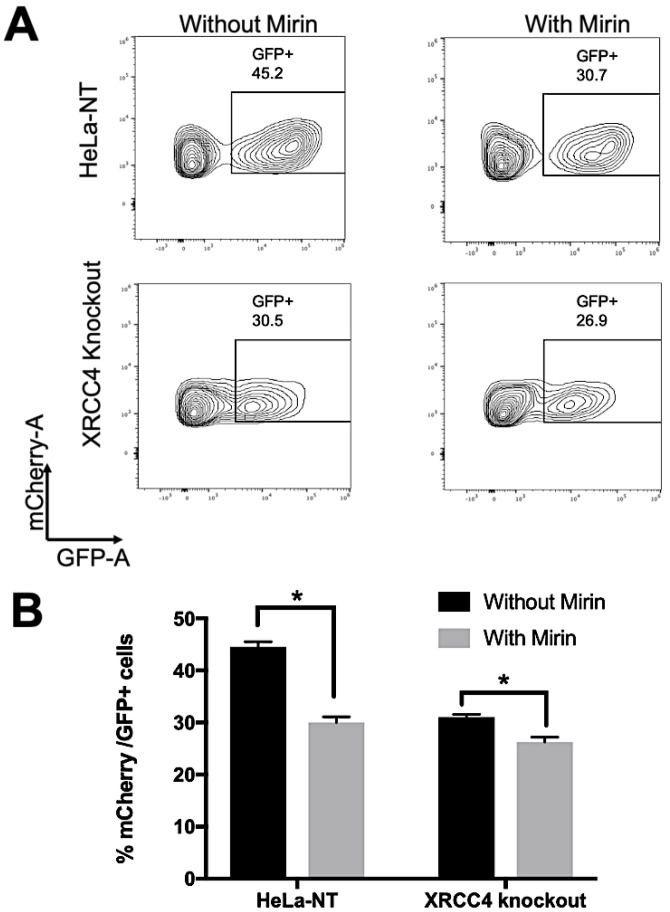
Measurement of repair efficiency using flow cytometry. (**A**) Representative images of cells positive for mCherry expression then gated for GFP^+^ expression. (**B**) Mean GFP^+^ as a percentage of mCherry^+^ cells were plotted with standard deviations. Experiments were performed in triplicate and data were analyzed using FLowJo 10.7.1. Statistical significance was determined by ANOVA where * indicates *p* < 0.01. All the experiments were performed independently three times and the data represent the mean ± standard deviation.

To quantify repair efficiency, cells positive for the fluorescent reporters were quantified by flow cytometry. Cells were first gated based on mCherry^+^ expression, selecting the population expressing the reporter system. Next, we determined the percentage of cells expressing GFP within the mCherry^+^ cell populations (Figure 3A,B). The mean percentage of GFP^+^ expressing cells within the mCherry^+^ cells for each sample is compared in Figure 3B. The relative efficiency of repair was (44.5% ± 1.0) in HeLa-NT cells and (30% ± 1.1) for HeLa-NT cells treated with mirin, when compared to control HeLa-NT cells (Figure 3B), indicating that blocking MRE11 exonuclease activity reduced repair efficiency. Similar inhibition of repair was observed in *XRCC4* knockout cells (31% ± 0.6) or those treated with mirin (26.3% ± 0.9). These results indicated that repair was possible even with a knockout of *XRCC4* or mirin treatment (inhibition of MRE11 exonuclease), or both in combination. The efficiency of repair as compared to control was reduced by ~30–40% in the various knockout and/or inhibited cell lines.

**Figure 4:**
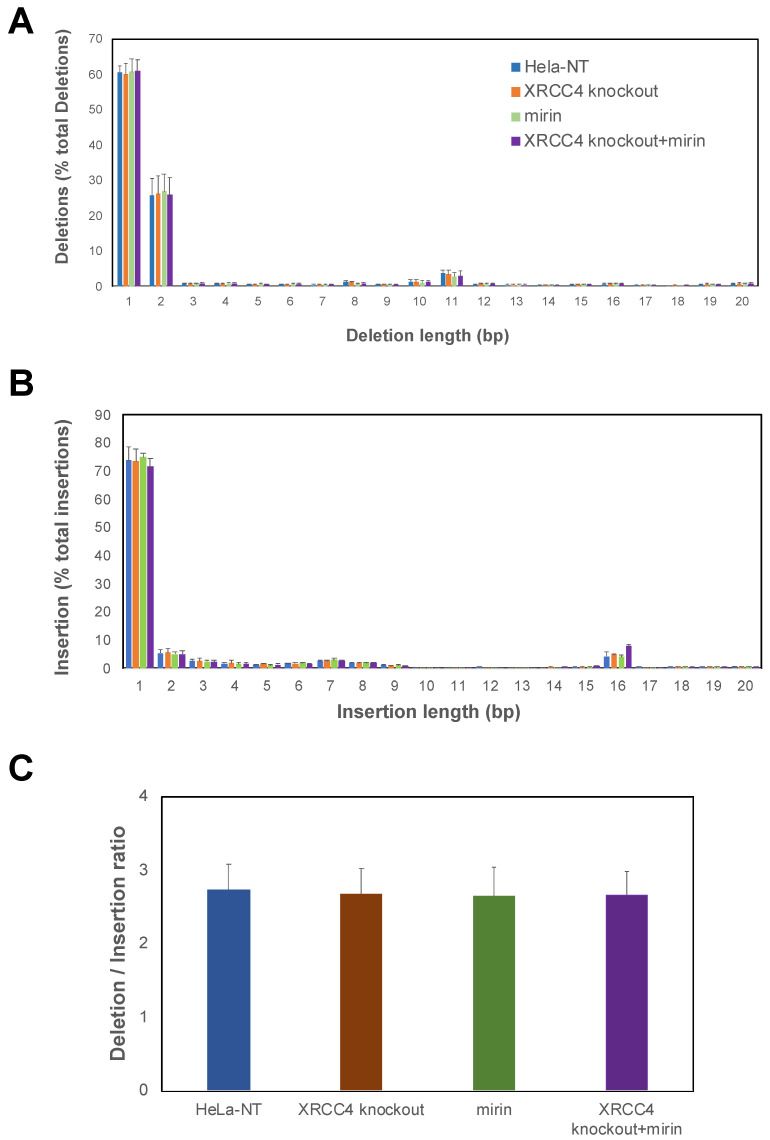
Editing profiles of HeLa cells with impaired repair pathways. Bar plots for the frequency of deletions (**A**) and insertions (**B**). Indel lengths are shown following DNA repair of TALEN-induced DNA damage. For each sample, we measured between 42,733 and 64,104 deletion and 19,598 and 25,620 insertion editing events. (**C**) Ratio of deletion to insertion Indels for HeLa cells with impaired repair pathways. Standard errors reflect duplicate measurements. The color key is in panel A.

We used NGS to determine if the impairment of repair pathways impacted the editing signatures in cells. We analyzed HeLa cells with impaired repair pathways by targeted sequencing of the TALEN-induced DNA damage region with NGS sequencing of DNA. For each sample, we analyzed ~75,000 total editing events. Deletions varied in length up to 20 bp, but were most commonly 1, 2, or 11 bps. Insertions were most frequently 1 bp, and less frequent for 2 or 15 bp. The ratio of deletions to insertions was ~3. There were no significant observed changes in the frequencies of the lengths of each indel (Figure 4A,B) or the ratio of deletions to insertions (Figure 4C) between any of the groups. These results indicate an extremely robust repair system where editing signatures remain resilient, even upon the impairment of proteins involved in major repair pathways. Furthermore, the editing signatures in the mirin alone, *XRCC4* knockout and *XRCC4* knockout + mirin-treated cells (Figure 3) were not significantly different from control. 

### 2.5. Altered Expression of Genes When Proteins Critical for Major Repair Pathways Are Blocked

**Figure 5:**
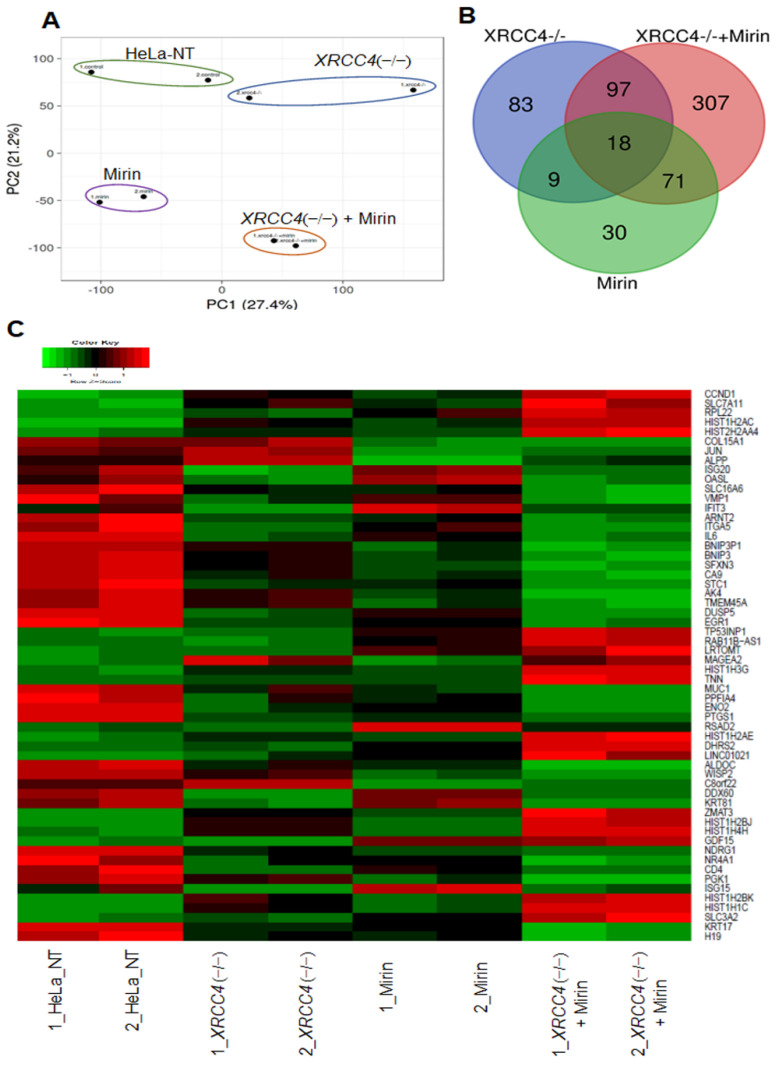
Principal component analysis (PCA), Venn diagram, and heatmap from RNA-seq profile. (**A**) A PCA plot for HeLa-NT, *XRCC4* knockout, mirin-treated, and *XRCC4* knockout + mirin-treated samples from whole-transcriptome RNA-seq data using ClusVis [32]. (**B**) A Venn diagram representing shared and unique differentially expressed genes (DEGs) across three categories: (i) *XRCC4* knockout cells (blue) (ii) mirin treated (green) (iii) *XRCC4* knockout with mirin treated (red) when compared to HeLa-NT cells. (**C**) A heatmap of top DEGs across each sample categories. Knockout = KO. The analyses are based on duplicate experiments.

The reporter assay demonstrates that DNA DSB repair is partially inhibited, but still present in *XRCC4* knockout cells and/or cells treated with mirin. To assess the molecular basis for the remaining repair activity, transcriptomes were compared for cells with *XRCC4* knockout plus and minus mirin treatment. mRNA from cells was sequenced by RNA-seq and transcripts were identified. We harvested RNA 48 h post-transfection to give enough time for the reporter and TALENs to be expressed, repair to occur or be blocked, and then enough time for longer-term transcriptional changes beyond that of typical intermediate-early genes. A principal component analysis showed a clear separation for the first two principal components between each sample category, but clustering for duplicate samples (Figure 5A), indicating a different transcriptomic profile for each sample category.

**Figure 6:**
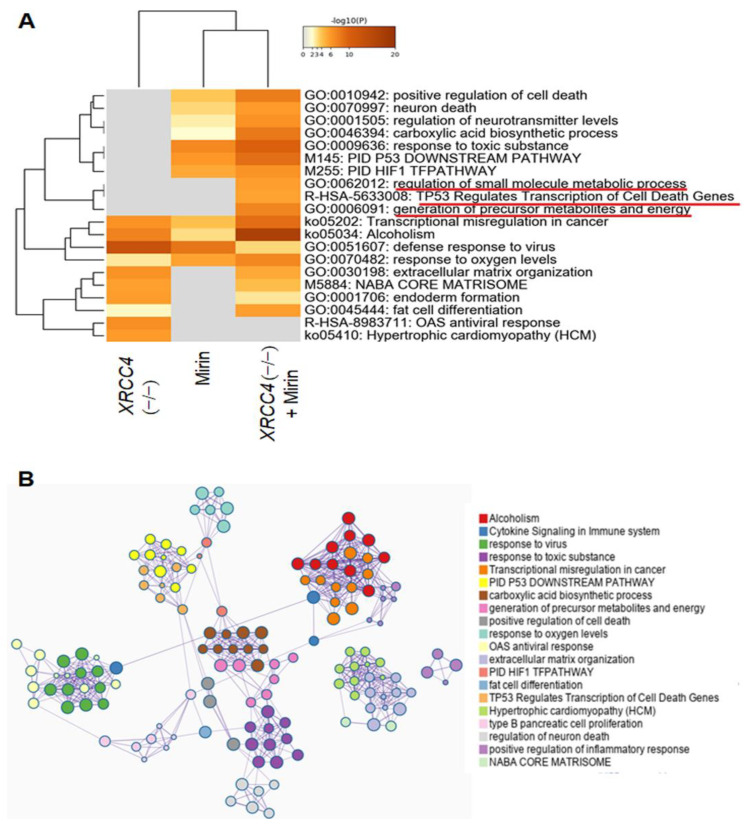
Functional enrichment analysis. (**A**) A heatmap with embedded dendrogram showing relationships between enriched GO/KEGG terms and canonical pathways. 0.3 kappa score was applied as the threshold to cast the tree into term clusters. Differential expression is calculated from samples compared to control HeLa-NT cells. Key pathways with large changes in gene expression for the *XRCC4* knockout +mirin sample are underlined with red lines. (**B**) A network graph with enrichment ontology clusters colored by cluster ID. Each term is represented by a node, where its size is proportional to the number of genes for each term. Terms with a similarity score >0.3 are linked by an edge (the thickness of the edge represents the similarity score). The network was created with Cytoscape (v3.1.2). The analyses are based on duplicate experiments.

The transcriptional changes are summarized in a Venn diagram to codify similar and unique genes among samples. The *XRCC4* knockout sample had 83 genes and mirin-treated HeLa cells had 30 unique DEGs (Figure 5B). However, the mirin-treated *XRCC4* knockout cells had 307 uniquely DEGs, far greater than other samples reflecting a more impactful transcriptional response. This differential transcriptional response was further supported when the top DEGs were compared with a heatmap (Figure 5C and Table S2). The gene expression for *XRCC4* knockout cells treated with mirin was the most different from HeLa-NT cells (Figure 5C). To validate the gene expression changes, five differentially expressed genes identified from the RNA-seq analysis (*CA9*, *CDKN1A*, *ENO2*, *DUSP5*, and *ZMAT3*) were assessed by real-time PCR. The expression levels of the top differentially expressed genes were plotted as a heatmap (Figure 5) and the p53 downstream pathway was selected for gene expression quantitation by real-time PCR (Figure 6A). The PCR data and RNA-seq gene expression measurements were consistent with each other, thereby validating the RNA-seq results (Figure S1, see Supplementary Materials). The cells with either *XRCC4* knockout or treated with mirin showed fewer and less intense changes in gene expression as compared to control cells. Additional details for changes in gene expression are shown by volcano plots in Figure S2.

### 2.6. Pathway Analysis of Differentially Expressed Genes (DEGs)

We next sought to determine which pathways are functionally associated with the transcriptional response to blocking major repair pathways. DEG enrichment was analyzed with Metascape [33]. Meta-enrichment analysis indicated that all three conditions with inhibited repair were consistently enriched in several pathways: transcriptional misregulation in cancer, alcoholism, defense response to viruses, and response to oxygen levels (Figure 6A). Pathways unique to *XRCC4* knockout cells with or without mirin were extracellular matrix organization, core matrisome, and endoderm and fat differentiation pathways (Figure 6A). Pathways such as carboxylic acid biosynthetic process, p53 downstream pathways, and HIF signaling pathway were enriched in *XRCC4* knockout + mirin group and HeLa-NT treated with mirin but more pronounced in *XRCC4* knockout + mirin group. Interestingly, pathways unique to *XRCC4* knockout with mirin group were regulation of small metabolic processes, P53 regulates transcription of cell death gene, generation of precursor metabolites and energy (Figure 6A). A network layout was created by enrichment analysis and visualized with Cytoscape (v3.1.2) (Figure 6B). Such a network reveals the interrelation of enriched pathways and genes.

### 2.7. Protein-Protein Interaction (PPI) Network Construction and Pathways Interaction Analysis

**Figure 7:**
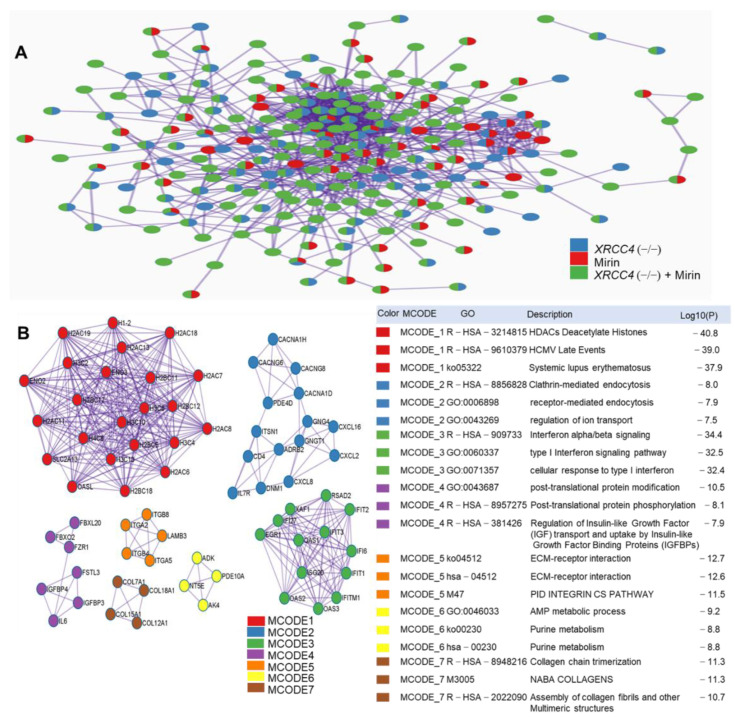
Protein–protein interaction network analysis. (**A**) PPI network of DEGs for each treatment group. Nodes are displayed as pies to indicate sample. (**B**) MCODE components were identified from the merged network for all samples. Each MCODE network is assigned a unique color and the network was generated with Cytoscape (v3.1.2). The MCODE GO term, description, and *p* values are shown. The analyses are based on duplicate experiments.

Pathways were also evaluated by examining protein–protein interaction networks. A protein–protein interaction network was constructed from the DEGs for the 3 sample conditions with BioGrid in Metascape [33]. The network contained a total of 237 nodes and 956 edges where network nodes are displayed as pies (Figure 7A). Seven significant network modules were identified with the MCODE algorithm (Figure 7B), which included 71 proteins from which *ENO3*, *CACNG6*, *ITGB8*, *PDE10A*, *COL12A1*, and *FSTL3* served as seed proteins. Gene Ontology (GO) terms associated with each module are depicted in Figure 7B and HDAC deacetylates histones appear to be the prominent GO term. Of relevance with the latter is that human histone acetylation and deacetylation have been shown to regulate the NHEJ repair pathways [34]. 

## 3. Discussion

We sought to better understand the cellular responses to TALEN-induced DSBs while eliminating or inhibiting the functions of two key DNA repair proteins XRCC4 and MRE11. First, we designed, tested, and validated a reporter construct that enabled us to examine whether the cells had undergone DNA repair. When *XRCC4* is knocked out or MRE11 exonuclease activity is inhibited, DNA repair was reduced by ~30%. Repair activity was reduced by ~40% in *XRCC4* knockout + mirin-treated cells as compared to wild type HeLa-NT cells. This observation indicates that repair of exogenous constructs is sustained, even when the functions of XRCC4 and exonuclease activity of MRE11 are abolished. These observations support the presence of backup repair pathways with redundant functions such as the A-EJ repair pathways [15]. Previous studies have noted that in DNA ligase IV (a protein that forms a complex with XRCC4) knock-out cell line, an increase in TALEN-induced deletions and insertions were observed suggesting a shift from NHEJ to alternative NHEJ (A-EJ) and microhomology-mediated EJ repair (MMEJ) [35]. Similarly, a compensatory response when pathways are inhibited or inactivated is supported by previous work in mouse cells with a knockout of *PRKDC*, the gene encoding DNA-PKcs and/or inhibition of RAD54 affecting NHEJ and HRR, respectively. This latter work revealed evidence of error-prone pathway(s) working to repair DSBs in the absence of the two major DSB repair pathways [15]. However, surprisingly, in the current study, the number and type of indels formed in the different repair defective cells were not significantly different between groups, suggesting at least with this exogenous construct, other repair pathways can repair these simpler breaks like controls. 

We considered three plausible explanations for the persistence and similarity of indels following DSB repair despite inhibiting XRCC4 and MRE11 exonuclease activity: (1) Involvement of some form of A-EJ repair, e.g., PolQ-dependent MMEJ repair [36] and (2) that other genes could compensate for functional deficits in MRE11 or XRCC4. Although Xing et al. identified *PAXX*, a new paralogue of *XRCC4* [37], this gene was not differentially expressed in our experiments; and (3) post-translational modifications of known DNA repair proteins or new protein-protein interactions that can modulate repair pathways would not be detected by the RNA-seq analysis. In addition, many DNA repair proteins are constitutively expressed, and not inducible upon damage exposure, thus modification of repair proteins, rather than extensive changes in transcript levels may be expected [38].

Considering the latter hypothesis, our DEG analysis identified multiple pathways that were induced in each of the inhibited populations. The results suggest induction of a substantial regulatory response that may produce signaling cross-talk between the repair pathways. Although, NHEJ is the major repair pathway for DSB in differentiated cells; the temporal recruitment of different factors and complexes are not completely elucidated. There may likely be interactions between these DNA repair pathways, which previous work suggests. 

In support of the latter hypothesis, studies on cross-talk of NHEJ with base excision repair (BER) and HDR with some common proteins were identified [39,40]. Xia et al. has demonstrated that Polβ which plays a central role in BER, exhibits a higher degree of spatial colocalization with Ku70, a component of NHEJ in the nucleus following DNA damage caused by methyl methanesulfonate or etoposide [41]. In vitro binding assays also support interaction between Polβ and Ku70. MRE11 has a critical role in DSB recognition and ATM recruitment in HRR pathways [42]. Another protein, BRCA1, is known to regulate the nuclease activity of MRE11. BRCA1 phosphorylation mediated by checkpoint kinase 2 (CHK2) enhances NHEJ fidelity but can also deplete 53BP1 in the S/G2 phase to favor HRR, suggesting that post-translation modification of BRCA1 regulates pathway preference [39,40]. Thus, it is possible that NHEJ could have signaling interactions from cross-talk that still allow repair [14,43]. 

We realize that the simple TALEN-induced DSB does not provide the same landscape of breaks that would be observed in a normal context in genomic DNA following damage. However, it was of interest to center on more uniform damage within a particular section of DNA to discern if indel patterns differed, thus predicting the unique usage of a variant repair pathway. However, between all groups of repair proficient and defective cells, there was a similar pattern of insertions and deletions overall, with insertions in nearly all cases just a single bp whereas the number of deleted nucleotides showed much more variability. The single base pair insertion observed is likely due to terminal deoxynucleotidyl transferase (TdT), which typically adds a single nucleotide of microhomology, and thus does not invoke the action of any polymerase in repairing this damage [44], whereas the wide variety of deletions exhibited perhaps reflect the differing A-EJ pathways utilized, whose repair may cause the elimination of various numbers of nucleotides depending on various factors, such as microhomology. The prevalent 11 bp deletion, again observed in all the four sample types, is also of interest. We expect that his unique type of edit is due to recursive cleavage by TALENs and repair of the DSB. Cycles of cleavage and repair may eventually produce a long enough deletion or insertion in the spacer region beyond which the Fok1 domains in the TALENs can no longer heterodimerize and cleave the DNA target region. Although one might expect differences in the types and ratios of various indels between repair defective and control, this may have been limited due to the uniform type of break within an exogenous construct which has been previously noted to not show the same patterns of repair pathway usage as compared to genomic DNA [45].

One theme that emerged was that blocking NHEJ pathways by either *XRCC4* knockout or mirin treatment alone or in combination lead to pathways such as transcriptional mis-regulation in cancer, alcoholism, defense response to viruses, and response to oxygen levels. The error-prone nature of NHEJ is a major cause of carcinogenesis and blocking of these pathways in our experiments correlates with the transcriptional mis-regulation associated with the cancer pathway. The introduction of DSB/viral DNA can activate DNA repair pathway components, which are also activated in a defense response to viruses [46]. Similarly, oxidative stress induces DNA damage and can activate repair [47]. Enrichment of these pathways highlights cellular adaptation in response to DNA damage. 

In interpreting these pathways, one must consider that RNA-seq is very sensitive, and mild changes in conditions for each sample such as DNA species during transfection could produce transcriptional changes. However, although the lead pathways must be verified by further investigation, the changes in the expression were generally repeatable among replicates, which is one approach to reducing false positives. Furthermore, we observed transcriptional differences for genes or pathways that would be expected or that make sense. For example, many histones had changes in expression (Figure 5C), and the p53 DNA damage response pathway and defense to virus pathways were changed; the latter can induce DSB during integration (Figure 6A). Furthermore, many genes were affected by more than one condition (Figure 5B).

Although, we did not identify any major DNA repair proteins to be uniquely altered in *XRCC4* knockout and mirin-treated cells, different pathways were enriched. For example, pathways associated with regulation of small-molecule metabolic processes, P53 regulates transcription of cell death genes and generation of precursor metabolites and energy, all suggesting a major role played by genes of metabolic pathways. The metabolic enzymes are known to play non-canonical roles outside their established metabolic roles especially in gene regulation, DNA damage response, and apoptosis [48]. Among the metabolic pathways, genes belonging to glycolysis were more prevalent and were downregulated. Induction of glycolysis contributes to the enhancement of NHEJ repair pathway and here we show that *XRCC4* knockout with mirin-treated cells had lower expression of glycolytic genes, *PGK1*, *ALDOC*, *PFKFB4*, *TPI1*, *ENO3*, *PFKP*, *ENO2*, *HK2*, when compared to control HeLa-NT cells [49]. However, whether there is a feedback loop mechanism between glycolysis and DNA repair pathway and its potential role in TALEN-induced damage repair will require further investigation. 

Depletion of another DNA repair enzyme, DNA-PKcs is previously shown to cause alteration in the metabolic pathway and P53 expression levels [50]. Enhanced p53 activity is a critical cellular signal for DNA damage. Under normal conditions, p53 remains in an inactivated state. However, in response to DNA damage, the p53 is activated and drives transcription of factors involved in the apoptosis, cell cycle, DNA repair, and cellular senescence [51,52]. Therefore, to compensate for defective XRCC4 and MRE11, our results show that the metabolic and p53 pathways are altered as an adaption to cellular stress introduced by DSB and in absence of efficient EJ repair systems. Previous work has also noted that inhibition of DNA PKcs improved HRR, suggesting a completion between pathways and that cells compensate and change the relative usage of repair pathways dependent upon the inhibition of pathway choice [53,54].

While the previously discussed p53 response has a well-known connection with DNA damage and repair, chromatin modifications may be required to give repair proteins access to the DSB. One major DEG category supports a possible role for chromatin modification as part of the regulator response to inhibiting repair. In support of this, a set of histones genes were identified in the protein–protein interaction studies, and these genes are also part of the alcoholism pathway that is highly enriched in the *XRCC4* knockout cells treated with mirin (Figure 7B). HDACs deacetylation histones have a major influence on chromatin structure and regulate the activation of DNA repair proteins [55]. Several reports are supporting the profiles of histone acetylation affecting NHEJ [56,57,58,59]. HDAC inhibitors impair DNA repair, suggesting that upregulation of HDAC deacetylation contributes to DNA repair in mirin-treated *XRCC4* knockout cells [60].

In summary, the protein–protein interaction network analysis results suggest interconnectivity between the transcriptional responses for *XRCC4* knockout and mirin treatment (Fig 7A). This is consistent with our hypothesis and prior evidence of cross-talk and competition between different DSB repair pathways. Due to the malleable nature of the repair pathways and the overlap in protein function in the various pathways, it is difficult to ascertain the exact pathway(s) activated; however, it is obvious, in the absence of *XRCC4* and inhibition of MRE11, that repair of exogenous DNA is robust and alternative pathways provide a similar spectrum of indels upon repair.

## 4. Materials and Methods

### 4.1. Repair Reporter Plasmid Construction

For construction of an EJ reporter plasmid, the sequence corresponding to a TALEN binding site (Figure 1A) was PCR amplified from pLai.2 HIV proviral plasmid (NIH AIDS Reagent Program #2532) and cloned upstream of the mCherry coding sequence into the NheI and AgeI sites of the pmCherry-C1 plasmid (Takara #632524). For cloning a TALEN binding site (TBS) downstream of the mCherry coding sequence, the TBS was amplified, fused with GFP coding sequence by overlap-PCR and cloned between the SalI and BamHI restriction enzyme sites. The coding sequence of EGFP was amplified from the plasmid pEGFP-C3. A spacer was introduced between the cytomegalovirus promoter (CMV) promoter and the upstream TBS at the NheI restriction enzyme site (New England Biolabs Inc., Ipswich, MA, USA) within the pmCherry-C1. Primers used for sequencing and cloning are listed in (Table S1).

### 4.2. Cell Culture and Lentivirus Production

HeLa (ARP 154) cells obtained from the NIH AIDS reagent program and LentiX293T cells (Clonetech Laboratories, Mountain View, CA, USA; #632180) were grown in Dulbecco modified Eagle’s Medium (DMEM) supplemented with 10% FetalClone III Serum (HyClone Laboratories, Logan, UT, USA; #SH30109.03). Lentivirus was produced from transfected LentiX293T cells. Briefly, 6 well plates were seeded with 0.6 million cells and incubated for 24 h prior to transfection. Cells were co-transfected with gXRCC4-lenticrisprV2/gScrambled-lenticrisprV2, 1.2 µg; psPAX-2 (Addgene Watertown, MA, USA; #12260), 1.0 μg, and pHEF-VSVG (Addgene #22501), 0.3 μg using the Lipofectamine LTX transfection reagent (Invitrogen, Waltham, MA, USA) at a 1:3 ratio [DNA (μg): Transfection reagent (μL)]. After 6 h of incubation, media was replaced, and cells were cultured in complete media for 48 h. Cell supernatant were collected, filtered through a 0.45 μm syringe filter (MilliporeSigma, Burlington, MA, USA), and used for transduction into HeLa cells. 

### 4.3. Assessment of DNA Editing

TALEN expression constructs (TAL 256 and TAL 278) were previously constructed with the Joung Lab REAL Assembly TALEN kit (Addgene, Watertown, MA, USA) [25,61]. To assess DNA repair, HeLa cells (0.6 million) were seeded a day before transient transfection, co-transfected with a mCherry/GFP reporter plasmid and pairs of TALEN-expressing constructs [TAL 256 (200 ng) and TAL 278 (200 ng)] at a ratio of 1:3 [DNA (μg): Transfection reagent (μL)] with Viafect transfection reagent (Promega, Madison, WI, USA). As a control, the reporter plasmid was co-transfected with empty vector [JDS70 (200 ng) and JDS 78 (200 ng)]. Medium was changed after 4 h incubation and replaced with complete medium with or without mirin (Sigma Aldrich, St. Louis, MO, USA; 100 μM). After 48 h, cells were tested for mCherry and GFP fluorescence by fluorescence microscopy and flow cytometry.

### 4.4. Generation of a XRCC4 Knockout Cell Line

To generate a *XRCC4* knockout HeLa cell line, a gXRCC4 sequence targeting the *XRCC4* gene (NCBI RefSeq:NC_000005.10) was annealed, phosphorylated with T4 Polynucleotide Kinase (New England Biolabs Inc., Ipswich, MA, USA) and cloned into the BsmBI digested LenticrisprV2 plasmid (Addgene, Watertown, MA, USA; #52961)). The plasmid was delivered into HeLa cells by lentiviral transduction. The cells were selected in complete media with puromycin (1.5 µg/mL) for 1 week followed by clonal selection. Clonal cells were screened for tri-allelic *XRCC4* knockout by Western Blotting with a XRCC4 antibody (Santa Cruz Biotechnology, Inc., Dallas, TX, USA; sc-271087) on Nitrocellulose membrane (GE Amersham, Waukesha, WI, USA) and by targeted sequencing of gDNA. One of the clones 2G3 (HeLa *XRCC4* knockout cells) was selected for all subsequent experiments. For the control HeLa cells, non-targeting guide RNA was similarly expressed in HeLa cells. Genomic DNA (gDNA) was isolated from HeLa *XRCC4* knockout and HeLa-NT (control cell containing non-targeting gRNA) using Quick -DNA MiniPrep Plus Kit (Zymo Research, Irvine, CA, USA). Isolated gDNA spanning the selected region of the *XRCC4* gene (NCBI RefSeq: NC_000005.10 (83104920-83105621) was amplified by PCR with XRCC4-SeqV2FP and RA-XRCC4seq-RP primers and Herculase II Fusion DNA polymerase (Agilent, Santa Clara, CA, USA). PCR amplified product was digested with XhoI (New England Biolabs, Ipswich, MA, USA), and subcloned into the XhoI and EcoRV restriction enzyme sites of the pBlueScript II SK (-) vector. Several colonies were screened for the *XRCC4* gDNA insert, and mutations in the *XRCC4* region in the 2G3 clonal cells were confirmed by sanger sequencing. 

### 4.5. Fluorescence Microscopy

Transfected cells were cultured for 48 h before measuring fluorescence. Live cells were assessed for EGFP and mCherry fluorescence in fresh media. Images were acquired at 200× magnification with a Nikon TE2000E epifluorescence microscope equipped with Photometrics CoolSNAP FX Camera (Roper Scientific, Sarasota, FL, USA). Images were captured in sequential scanning mode to avoid spectral bleed through and were analyzed in triplicate with multiple scanning regions for each.

### 4.6. Fluorescent Activated Cell Sorting (FACS) 

Transfected cells were cultured for 48 h before flow cytometry analysis. Cells were trypsinized, washed twice with phosphate buffered saline (PBS) and fixed with 2% paraformaldehyde for 10 min at RT. Cells were washed twice with PBS before acquisition with a flow cytometer (Sony SH800, San Jose, CA, USA). Cells were gated first for mCherry expression followed by GFP expression. Experiments were analyzed using FlowJo 10.7.1 for triplicate samples.

### 4.7. RNA-seq and Data Analysis

Cells were transfected with a mCherry/GFP reported plasmid and TALEN constructs and treated with mirin or vehicle control after 4 h post-transfection. Cells were harvested 48 h after transfection and total RNA was extracted with the Quick-RNA Miniprep Plus Kit (Zymo Research, Irvine, CA, USA). Duplicate samples for each condition were analyzed. RNA was quantified, RNA-seq was performed at a depth of more than 20 million paired end reads for each sample (Novogene, Cambridge, UK).

The RNA-seq data can be accessed at Gene Expression Omnibus (GEO) (accession no GSE135274). For RNA-seq analysis, raw reads were imported into CLC Genomics Workbench 12.0 and trimmed using the quality limit score of 0.05 calculated from a modified-Mott trimming algorithm [62], read through adapter trimming, and trimming of ambiguous bases from read regions with more than two ambiguous reads [62]. RNA-seq analysis was performed with the RNA-seq Analysis tool in Genomics Workbench using default settings including a mismatch cost of 2 with insertion and deletion cost of 3. The Reference genome was hg19 and reference gene track used was ensemble_v74. Differential expression analysis was performed in Genomics Workbench with the Identify and Annotate Differentially Expressed Genes (DEGs) software. 

### 4.8. DNA Editing Indel Analysis

Cells were transfected as in Section 4.2. Post-48 h transfection, the plasmids from the transfected cells are isolated using the Zyppy plasmid miniprep kit (Zymo Research, Irvine, CA, USA). The region spanning the edited region within the plasmid was amplified using the Herculase polymerase (Agilent, Santa Clara, CA, USA) with primers (TalIFP NheI and mCherry Tal1RP). DNA libraries were prepared from the PCR amplified DNA (100 ng) using NEBNext Ultra DNA Library prep kit for Illumina (New England Biolabs Inc., Ipswich, MA, USA) and the manufacturer’s protocol. Briefly, the DNA library preparation involved multiple steps that include end repair and adaptor ligation. The size exclusion selection and DNA clean-up after every step was performed using the 2× volume of Ampure XP beads (Beckman Coulter, Brea, CA, USA). The DNA with ligated adaptors DNA was further enriched by PCR (6 cycles) using the Universal PCR Primer/i5 primer and Index Primer/i7 Primer of the NEBNext Muliplex Oligos for Illumina (New England Biolabs Inc., Ipswich, MA, USA). Post-cleanup with 2X Ampure XP beads, the samples were quantified and analyzed for size distribution using Agilent bioanalyzer 2100 (Agilent). The DNA library was denatured and mixed with 20% denatured ΦX174. The samples were sequenced in NextSeq500 (Illumina, San Diego, CA, USA). DNA-seq was performed at a depth of more than 1 million paired end reads for each sample (Novogene, Cambridge, UK). Reads were aligned and the length and frequencies of Indels were quantified. 

### 4.9. Bioinformatic Analysis

A principal component analysis (PCA) of RNA-seq data and resulting plot were created with ClustVis accessed on 24 February 2021 [32]. Venn Diagrams of differentially expressed genes for each condition was compared to control (FDR < 0.01 log FC >= 1) and plotted with an online tool (http://bioinformatics.psb.ugent.be/webtools/Venn/ accessed on 13 January 2021. Heatmaps for the DEGs were plotted using the Heatmap.2 function in the R ggplot package R [63]. Volcano plots for each category were created using “EnhancedVolcano” function in R [64]. Gene enrichment analysis was performed for DEGs lists (FDR < 0.01 and logFC > 1.2) with Metascape [33]. A network graph was created with Cytoscape (v3.1.2). Term relationships having a similarity score above 0.3 are connected by edges. The network is visualized with Cytoscape (v3.1.2).

For indel analysis, raw reads were trimmed for capturing TALEN editing inserts/amplicons and removing TALEN binding primer sequences (5′-primer: GAAGAAGCGTAAGGTCTAGC and 3′-primer: TAACTAGGGAACCCACTGCT) using Cutadapt [65]. Read 1 and read 2 from pairs were processed independently as technical replicates, as it was unnecessary to consider multiple alignments here. Trimmed reads with TALEN editing inserts were aligned to the reference sequence (GAGCCTGGGAGCTCTCTGGC) using BWA-MEM [66]. Nucleotide substitutions, insertions and deletions introduced by TALEN editing were identified/called as variants (SNVs and Indels) using SAMtools/BCFtools mpileup [67] and summarized using modified parameters (–max-dept ReadCount) to break through maximum read depth (default: 250). Finally, called variants (.vcf file) from each sample were summarized using a Perl script and imported into an Excel file for comparison of indel lengths.

### 4.10. Protein–Protein Interaction (PPI) Network Analysis and Pathway Interrelation Analysis

PPI networks were constructed using multiple DEGs list based on BioGrid, InWeb_IN and OmniPath database in Metascape. For networks which contain between 3 and 500 proteins, Molecular Complex Detection (MCODE) algorithm was used to identify densely connected network components with default parameters. For each MCODE component, pathway and process enrichment analysis was applied and the three best-scoring (by *p* value) terms were retained as the functional description of the resulting modules. Resulting network graphs were visualized through Cytoscape (v3.1.2).

### 4.11. Quantitative Real-Time Polymerase Chain Reaction (qRT-PCR)

For validation of RNA-seq results, cells were transfected with a mCherry/GFP EJ reporter plasmid, TALEN expression constructs, and treated with mirin or vehicle control as described above. Cells were harvested 48 h after transfection, Total RNA was extracted using the Quick-RNA Miniprep Plus Kit (Zymo Research, Irvine, CA, USA) and cDNA was synthesized with SuperScript™ IV VILO™ Master Mix (Invitrogen-Thermo Fischer Scientific, Waltham, MA, USA). Gene expression of select DEGs were quantified by qRT-PCR with gene-specific primers (Table S1), and PowerUp SYBR Green PCRmix (Invitrogen-Thermo Fischer Scientific) in a Bio-Rad CFX96 Touch™ Real-Time PCR Detection System. Relative expression levels were normalized to a housekeeping control gene, *β-actin*. The fold changes in mRNA levels between the HeLa-NT and the experimental condition were calculated using the 2^−(ΔΔCT)^ method.

### 4.12. Statistical Analysis

Statistical analyses were conducted with Student’s *t*-test or by ANOVA for comparing more than 2 groups and a *p*-value ≤ 0.05 was considered significant. 

## 5. Conclusions

In conclusion, our results demonstrate that repair of TALEN-induced DS breaks is robust even when two key DNA repair proteins involved in the major DSB pathways are blocked or inhibited. The majority of repair activity was preserved with a corresponding transcriptional response. In the absence of both XRCC4 and inhibition of MRE11, new transcriptional responses likely associated with sub-pathways of A-EJ are evident.

## Data Availability

All data generated and analyzed during this study are included in this manuscript and its supplementary information files. RNA-seq files were deposited in the GEO (Gene expression omnibus) database. The accession number is GSE135274.

## Supplementary Materials

The following are available online at https://www.mdpi.com/article/10.3390/ijms23020593/s1.
