# Supplementary material for: XRCC4 and MRE11 Roles and Transcriptional Response to Repair of TALEN-Induced Double-Strand DNA Breaks
Source: Int J Mol Sci. 2022 Jan 6;23(2):593. doi: 10.3390/ijms23020593 (PMC8776116; doi:10.3390/ijms23020593)
Supplement: Supplementary file 1 [file ijms-23-00593-s001.zip › ijms-1522393-supplementary.pdf]

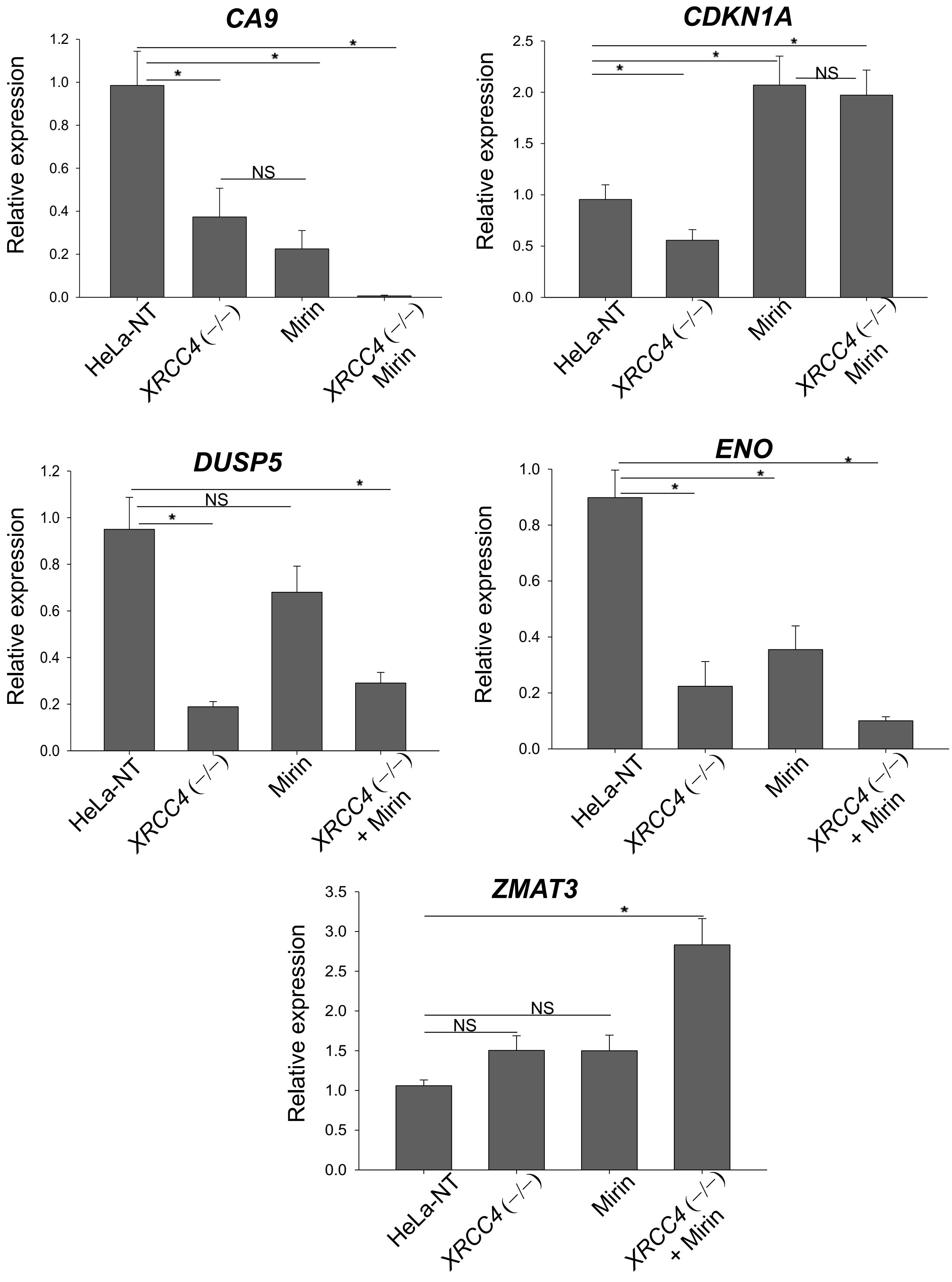

Supplementary Figure S1: Validation of RNA-seq profile across all the categories.
Changes in gene expression as analyzed by real time PCR. The data were represented as mean ± SD and significance between groups were determined by ANOVA test where *denoted $p < 0.05$ and NS=Not significant.

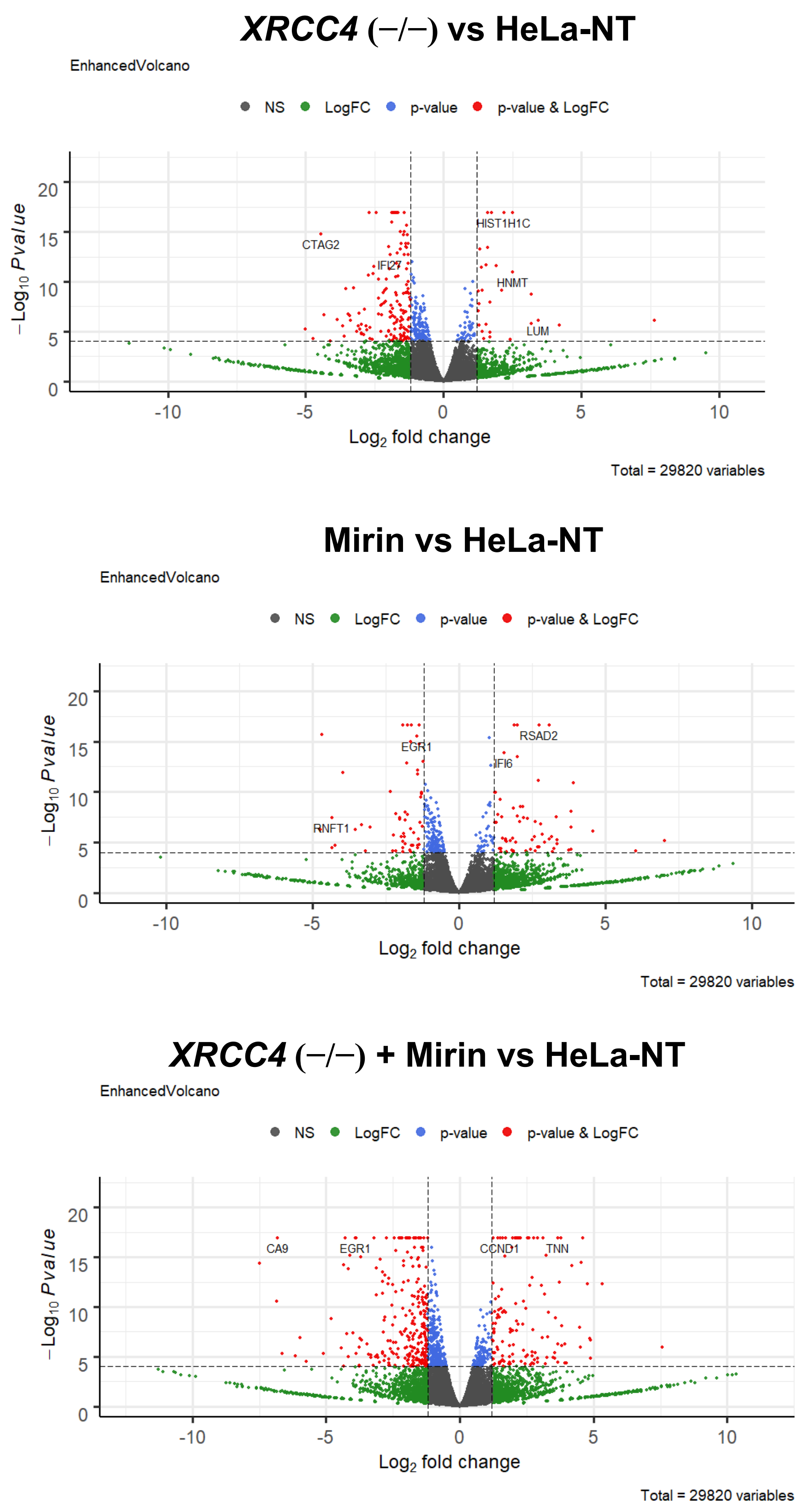

Supplementary Figure S2: Volcano chart and functional enrichment analysis. Volcano chart showing differentially expressed genes with FDR<0.01 and logFC>=1.2 compared to HeLa-NT cells A) *XRCC4*(−/−), B) Mirin treatment, C) *XRCC4(−/−)* + Mirin treatment.
